# Factors associated with lower forced vital capacity in children and adults with Duchenne muscular dystrophy using non-invasive ventilation: a multicenter analysis

**DOI:** 10.1007/s11325-024-03183-1

**Published:** 2025-01-07

**Authors:** Kanokkarn Sunkonkit, Manju Hurvitz, Andrew Defante, Jeremy Orr, Abhishek Chakraborty, Reshma Amin, Rakesh Bhattacharjee

**Affiliations:** 1 Division of Respiratory Medicine, The Hospital for Sick Children, University of Toronto, Toronto, Canada; 2 Division of Pulmonary and Sleep Medicine, Department of Pediatrics, Faculty of Medicine, Chiang Mai University, Chiang Mai, Thailand; 3 Division of Respiratory Medicine, Department of Pediatrics, University of California San Diego, Rady Children’s Hospital, 9500 Gillman Drive, La Jolla, CA 92093 USA; 4 Division of Pulmonary and Critical Care Medicine, Department of Medicine, University of California San Diego, San Diego, CA USA; 5 Columbia University, New York, NY USA

**Keywords:** Duchenne muscular dystrophy, Forced vital capacity, Non-invasive ventilation, Socioeconomic determinants, Healthcare service determinants

## Abstract

**Background:**

Reduced forced vital capacity (FVC) is associated with morbidity and mortality in individuals with Duchenne muscular dystrophy (DMD). Non-invasive ventilation (NIV) is often prescribed for the treatment of sleep-disordered breathing (SDB), and chronic respiratory insufficiency. Despite the common practice of initiating NIV later in the progression of DMD, the factors influencing FVC subsequent to the commencement of NIV remain unclear.

**Objective:**

To evaluate the demographic, clinical and socioeconomic determinants of FVC% predicted across several cohorts of DMD children and adults prescribed NIV.

**Methods:**

A multicenter retrospective review of individuals with DMD prescribed NIV was performed between February 2016 to October 2020. Patients were identified from three sites: The Hospital for Sick Children, Canada; Rady Children’s Hospital San Diego, USA; and University of California San Diego Health, USA. Multivariate regression analysis was performed to determine factors that influence FVC.

**Results:**

Fifty-nine male patients with DMD prescribed NIV (mean ± SD for age and BMI was 20.1 ± 6.7 years and 23.8 ± 8.8 kg/m^2^) were included. Following multivariate analysis, a lower FVC% predicted was associated with older age (β = -1.44, *p* = *0.001*), presence of scoliosis (β = -16.94, *p* = *0.002*), absent deflazacort prescription (β = 14.43, *p* = *0.009*), and use of in-ex sufflator (β = -39.73, *p* < *0.001*), respectively.

**Conclusion:**

In our study, several factors were associated with reduced FVC% predicted in a DMD population using NIV. Future, prospective, longitudinal studies are imperative to comprehend the trajectory of FVC% predicted over time in individuals with DMD using NIV.

## Introduction

Duchenne muscular dystrophy (DMD) is an X-linked recessive neuromuscular disorder manifested by the absence or deficiency of functional dystrophin protein in muscle resulting in chronic inflammation, replacement of muscle with fibrotic and fatty tissue and resulting muscle weakness [1, 2]. Respiratory complications remain as a major contributor to morbidity and mortality in individuals with DMD due to progressive loss of muscle strength. The consequences of progressive muscle weakness, in particular the muscles of respiration, leads to restrictive respiratory disease, scoliosis and burden of respiratory disease including ineffective cough, atelectasis, sleep-disordered breathing (SDB) and respiratory failure [3–8]. These complications are precluded by respiratory function surveillance [3]. Routine assessment of pulmonary status typically initiated in late in the first decade of life is a fundamental component of the prevailing standard of care for patients with DMD including regular pulmonary function testing (PFT) [3], respiratory muscle strength and polysomnography (PSG) to screen for SDB [5, 6, 9]. SDB is often managed with non-invasive ventilation (NIV) [5, 6]. Furthermore, optimal evaluation of airway clearance techniques, regular chest physiotherapy and vaccination are also recommended to mitigate the risk of recurring pulmonary infections in patients with DMD [5, 6, 10].

Forced vital capacity (FVC) is the best global assessment of respiratory muscle strength [11]. FVC less than 1 L is associated with mortality [12]. Factors associated with FVC include steroid therapy including deflazacort [13–16], age [15], age of loss ambulation [17] and early use of NIV [18] have been described. NIV in particular, aids in maintaining optimal ventilation, reducing respiratory work of breathing, and preventing respiratory muscle fatigue. NIV has a positive impact on gas exchange, respiratory mechanics, and patient outcomes, highlighting its efficacy in improving pulmonary function [19]. Notwithstanding, recent question as to whether NIV reduces decline in FVC% predicted has come into question, particularly in patients using corticosteroids [18]. The complexity of this issue emphasizes the need for a nuanced approach to address the multifaceted challenges associated with pulmonary function in DMD patients.

Using a multicenter approach, our goal was to more precisely delineate the factors influencing pulmonary function in patients with DMD subsequent to the initiation of NIV. The aims of our study were to 1) evaluate FVC across a pediatric and adult cohort of individuals with DMD prescribed NIV and 2) identify clinical and socioeconomic determinants of FVC.

## Materials and methods

### Study design and setting

We performed a multicenter, retrospective study of patients with DMD followed at 1) The Hospital for Sick Children (SickKids), Toronto, Ontario, Canada 2) Rady Children’s Hospital (RCHSD), San Diego, California, USA and 3) University of California San Diego Health (UCSD), San Diego, California, USA.

This study was approved by the Research Ethics Board at The Hospital for Sick Children, Toronto, Canada (REB No. 1000064481) and University of California San Diego Health, San Diego, USA (REB No. 190155). As this is a retrospective study with minimal risk for patients, the requirement of informed consents was waived by the Research Ethics Board.

The electronic patient records from the Long-Term Ventilation clinic at The Hospital for Sick Children, Toronto, Canada and the Sleep Clinics at Rady Children’s Hospital, San Diego, USA and University of California San Diego Health were retrospectively reviewed between February 1, 2016 and October 1, 2020.

### Study population

#### Eligibility criteria

- *Inclusion Criteria:* 1) Male children and adults diagnosed with DMD through genetic testing, who have been prescribed NIV, such as bi-level positive airway pressure (Bi-level PAP) or volume-assured pressure support (VAPS). 2) PFT completion during the study period.
- *Exclusion Criteria*: 1) patients with DMD who were unable to perform adequate PFTs during the study period; 2) patients not prescribed NIV.

### Data collection methods

#### Demographics and medical history

We collected study participant information including age, age of DMD diagnosis, age at loss of ambulation, height, weight, body mass index (BMI) (calculated as weight (kg)/height (m)^2^), ethnicity, primary language, number of caregivers in the home, comorbidities, medications, ambulatory status, presence of lower respiratory tract infection (LRTI), rates of hospitalization for LRTI within the past 12 months, history of Emergency Department (ED) or clinic visit due to LRTI within the past 12 months, history of antimicrobial drugs use for LRTI within the past 12 months, history of surgery (such as scoliosis injury and/or adenotonsillectomy (AT)), medical technology at home (eg. in-ex sufflator, wheelchair and high frequency chest wall oscillator (HFCWO)), PFT and respiratory muscle strength measurements. We also reported household income. For Canadian patients we determined median household income using forward sortation area data by Statistics Canada [20]. We then determined if the household income was above or below the low-income cutoff before taxes by Statistics Canada [20]. For American patients, median household income was inferred from demographic zip code using the 2018 United States Census Bureau report for household income [21, 22].

#### Pulmonary function testing

##### The hospital for sick children

Spirometric data was performed using the laboratory spirometry system by Vmax Encore System. Daily calibration according to American Thoracic Society (ATS)/ European Respiratory Society (ERS) recommendations was performed on the conventional spirometers [23] and volume accuracy verification was performed on the handheld turbine spirometers before use. These tests use consistent coaching techniques as per ATS/ERS standards [23]. Indices obtained included forced expiratory volume in 1 s (FEV_1_), FVC, the ratio of FEV_1_ to FVC (FEV_1_/FVC), flows at lower lung volumes (FEF_25%‐75%_), and peak expiratory flow (PEF). Measurements that did not meet ATS standards for acceptability and reproducibility were rejected. Percent predicted values were obtained based on Global Lung Initiative normative data [24]. All patients completed seated PFT. Height was measured while standing, but for patients who could not stand, arm-span or ulnar length was measured and used as a substitute for height [25, 26].

##### Rady Children’s Hospital and University of California San Diego Health

All patients underwent seated PFT. Ulnar length was used to estimate height in patients with medical conditions that precluding obtaining height measurement directly. PFT were performed using C-NHANES III reference values and ATS criteria for acceptability and repeatability [23]. PFT parameters were evaluated at the time of the neuromuscular clinic visit or the most recent (< 6 months) neuromuscular clinic visit and included percent predicted and absolute value for seated FVC and FEV_1_. All patients underwent seated PFT.

### Statistical analysis

The data of individuals with DMD were summarized using descriptive statistics. Baseline characteristics, socioeconomic determinants, medical history and PFT data were reported as mean (standard deviation; SD) for normally distributed continuous variables and as median (interquartile; IQR) and frequency (percent) for skewed continuous variables and categorical variables, respectively. Normally distributed, continuous variables were compared using student’s t-tests and categorical variables were compared using Chi-square test. Factor associated with FVC% predicted in individuals with DMD who were prescribed NIV were analyzed using bivariate and multivariable regression analysis. Clinically relevant variables which were determined to be statistically significant following univariate analysis were then analyzed by multivariate regression analysis. A *p*-value less than 0.05 indicated statistical significance. Data analysis was carried out using R version 4.0.3.

## Results

Fifty-nine male children and adults with DMD using NIV for SDB were included. Overall, the mean ± SD for age and BMI was 20.1 ± 6.7 years and 23.8 ± 8.8 kg/m^2^, respectively. Mean ± SD age of diagnosis and age of loss of ambulation were 5.0 ± 2.6 years and 10.4 ± 2.4 years, respectively. Most patients were Non-Hispanic (*n* = 36, 61.1%), English was the primary spoken language at home (*n* = 51, 86.4%), had 2 or more caregivers in the home (*n* = 43, 72.9%), and had a household income above the low-income cut-off (n = 32, 54.2%) There were site specific differences based on SickKids, RCHSD and UCSD in demographic factors (Table 1).

**Table 1:** Demographic factors of patient with DMD prescribed NIV by site (*n* = 59)

Parameters		SickKids (N = 26)	RCHSD (N = 13)	UCSD (N = 20)	Overall (N = 59)	*p*-value
Age (years)		15.4 ± 1.8^*,⍬^	17.2 ± 3.9^⍦^	28.0 ± 4.6	20.1 ± 6.7	< *0.001*^*#*^
Age at diagnosis (years)		4.9 ± 2.0	4.0 ± 3.4	6.1 ± 2.8	5.0 ± 2.6	NS
Age at loss of ambulation (years)		11.2 ± 2.8^*^	8.8 ± 2.13^⍦^	10.3 ± 2.5	10.4 ± 2.4	*0.005*^*#*^
Body mass index (kg/m^2^)		24.4 ± 5.4^⍬^	24.7 ± 12.2^⍦^	22.5 ± 10	23.8 ± 8.8	NS
Ethnicity	Hispanic, n (%)	1 (3.8)^*, ⍬^	10 (76.9)	12 (60.0)	23 (38.9)	< *0.001*^*#*^
	Non-Hispanic, n (%)	25 (96.2)^*, ⍬^	3 (23.1)	8 (40.0)	36 (61.1)	
Primary Language	English, n (%)	25 (96.2)	9 (69.2)	17 (85.0)	51 (86.4)	NS
	Non-English, n (%)	1 (3.8)	4 (30.8)	3 (15.0)	8 (13.6)	
Number of Caregivers	1 Caregiver, n (%)	3 (11.5)^*^	9 (69.2)^⍦^	4 (20.0)	16 (27.1)	< *0.001*^*#*^
	2 Caregivers or more, n (%)	23 (88.5)^*^	4 (30.8)^⍦^	16 (80.0)	43 (72.9)	
Household Income	High income, n (%)	25 (96.2)^*, ⍬^	3 (23.1)	4 (20.0)	32 (54.2)	< *0.001*^*#*^
	Low income, n (%)	1 (3.8)^*, ⍬^	10 (76.9)	16 (80.0)	27 (45.8)	
Insurance Type	Private, n (%)	0 (0.0)^⍬^	3 (23.1)	5 (25.0)	8 (13.6)	*0.026*^*#*^
	Government, n (%)	26 (100.0)^⍬^	10 (76.9)	15 (75.0)	51 (86.4)	

Data presented as mean ± SD

^#^denotes *p*-value < 0.05 when comparing all institutions; NS denotes statistically non-significant *p*-value of ≥ 0.05; * denotes *p*-value < 0.05 when comparing SickKids to RCHSD; ⍬ denotes *p*-value < 0.05 when comparing SickKids to UCSD; ⍦ denotes *p*-value < 0.05 when comparing RCHSD to UCSD. *DMD, *duchene muscular dystrophy;* NIV, non-invasive ventilation; RCHSD, Rady Children’s Hospital, San Diego, CA, USA; SickKids, The Hospital for Sick Children, Toronto, Canada; UCSD, University of California Health, San Diego, CA, USA*

Table 2 demonstrates the clinical characteristics and healthcare service utilization across the three different institutions. Overall, mean ± SD of FVC, FEV_1_ and FEV_1_/FVC were 41.6 ± 31.0%, 42.0 ± 30.0% and 95.8 ± 18.3% predicted value, respectively. Spirometry parameters were different based on different institutes including FVC(%) (SickKids 66.0 ± 27.3; RCHSD 32.9 ± 19.5; and UCSD 15.1 ± 9.1, *p* < *0.001*); FEV_1_(%) (SickKids 65.2 ± 26.0; RCHSD 35.2 ± 21.0; and UCSD 15.9 ± 8.9, *p* < *0.001*); and FEV_1_/FVC (SickKids 97.2 ± 4.2; RCHSD 86.9 ± 22.2; and UCSD 99.2 ± 25.0, *p* = *0.005*), respectively. Deflazacort prescription (SickKids n = 24, 92.3%; RCHSD n = 6, 46.2%; and UCSD n = 3, 15.0%, *p* < *0.001*) varied significantly between institutions. However, the presence of LRTI within the past 12 months preceding rates of hospitalization for LRTI and ED/Clinic visits related to LRTI were not different. Medical equipment utilization was also similar between institutions for in-ex sufflator (SickKids n = 20, 76.9%; RCHSD n = 13, 100%; and UCSD n = 20, 100%) and wheelchair (SickKids n = 26, 100%; RCHSD n = 12, 92.3%; and UCSD n = 20, 100%) use but varied between Canadian and American Institutions with regards to HFCWO (Canadian n = 0, 0%; and American n = 5, 15.2%, *p* < *0.05*).

**Table 2:** Clinical characteristics and healthcare service utilization of the study cohort (*n* = 59)

Parameters	SickKids (*N* = 26)	RCHSD (*N* = 13)	UCSD (*N* = 20)	Overall (*N* = 59)	*p*-value
FVC (%)	66.0 ± 27.3^*,^^⍬^	32.9 ± 19.5^⍦^	15.1 ± 9.1	41.6 ± 31.0	< 0.001*
FEV_1_(%)	65.2 ± 26.0	35.2 ± 21.0	15.9 ± 8.9	42.0 ± 30.0	< 0.001*
FEV_1_/FVC (%)	97.2 ± 4.2^*^	86.9 ± 22.2^⍦^	99.2 ± 25.0	95.8 ± 18.3	0.005*
LVEF (%)	55.1 ± 13.2^⍬^	51.4 ± 10.8	44.6 ± 10.6	51.2 ± 12.6	0.014*
Presence of scoliosis, n (%)	12 (46.2)^*,^^⍬^	12 (92.3)	19 (95.0)	43 (72.9)	0.006*
Deflazacort prescription, n (%)	24 (92.3)^*,^^⍬^	6 (46.2)	3 (15.0)	33 (55.9)	< 0.001*
Presence of LRTI within the past 12 months, n (%)	4 (15.4)	4 (30.8)	4 (20.0)	12 (20.3)	NS
Hospitalized for LRTI, n (%)	3 (11.5)	5 (38.5)	5 (25.0)	13 (22.0)	NS
ED Visit for LRTI, n (%)	3 (11.5)	4 (30.8)	4 (20.0)	11 (18.6)	NS
Clinic Visit for LRTI, n (%)	1 (3.8)	0 (0.0)	1 (5.0)	2 (3.4)	NS
Antimicrobial prescription for LRTI, n (%)	4 (15.4)^⍬^	6 (46.2)	11 (55.0)	21 (35.6)	0.018*
Use of in-ex sufflator, n (%)	20 (76.9)	13 (100)	20 (100)	53 (89.8)	0.014*
Use of wheelchair, n (%)	26 (100)	12 (92.3)	20 (100)	58 (98.3)	NS
Use of HFCWO, n (%)	0 (0.0)^*^	4 (30.8)	1 (5.0)	5 (8.5)	0.003*

Data presented as mean ± SD

^*^ denotes *p*-value < 0.05 when comparing SickKids to RCHSD; ⍬ denotes *p*-value < 0.05 when comparing SickKids to UCSD; ⍦ denotes *p*-value < 0.05 when comparing RCHSD to UCSD. *DMD, duchene muscular dystrophy; ED, emergency department; FEV*_*1*_*, forced expiratory volume in 1-s; FEV*_*1*_*/FVC, ratio of forced expiratory volume in 1-s to forced viral capacity; FVC, percent predicted forced vital capacity; HFCWO, high frequency chest wall oscillator; LRTI, lower respiratory tract infection; LVEF, left ventricular ejection fraction; NIV, non-invasive ventilation; RCHSD, Rady Children’s Hospital, San Diego, CA, USA; SickKids, The Hospital for Sick Children, Toronto, Canada; UCSD, University of California Health, San Diego, CA, USA*

**Fig. 1:**
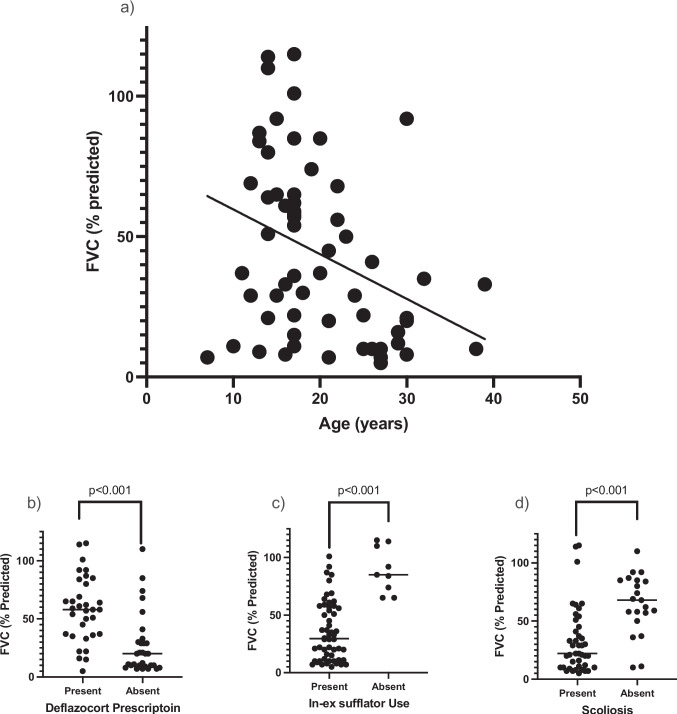
Univariate analysis of the relationship between forced vital capacity ( FVC % Predicted) and: **a**) Age (r.^2^ = 0.12, *p* < 0.001). **b**) Deflazacort prescription (*p* < 0.001). **c**) In-ex sufflator use (*p* < 0.001). **d**) Presence of scoliosis (*p* < 0.001)

See Table 3 for the univariate and multivariate results. According to multivariate analysis, the only factors significantly associated with FVC% predicted were age (β = −1.44, *p* = *0.001*), presence of scoliosis (β = −16.94, *p* = *0.002*), deflazacort prescription (β = 14.43, *p* = *0.009*), and in-ex sufflator use (β = −39.73, *p* < *0.001*), respectively. The relationship of these four variables with FVC% predicted is depicted in Fig. 1.

**Table 3:** Factors associated with FVC % predicted in individuals with DMD using NIV (n = 59)

Parameters	Univariate analysis			Multivariate analysis			
	B coefficient	SE	*p*-value	B coefficient	SE	95%CI	*p*-value
Age (years)	−3.03	0.46	< 0.001*	−1.44	0.42	−2.28 to −0.61	0.001*
Presence of Scoliosis	−29.36	7.97	< 0.001*	−16.94	5.22	−27.42 to −6.46	0.002*
Deflazacort prescription	35.08	6.80	< 0.001*	14.43	5.39	3.61 to 25.26	0.009*
Use of in-ex sufflator	−56.42	11.19	< 0.001*	−39.73	7.84	−55.47 to −23.99	< 0.001*
Insurance (public/government)	30.55	11.19	0.008*	12.41	6.79	−1.21 to 26.05	0.073

^*^ *p* ≤ 0.05 represents a statistically significant difference; *DMD, duchene muscular dystrophy; FVC, percent predicted forced vital capacity; NIV, non-invasive ventilation*

## Discussion

DMD is a progressive degenerative neuromuscular disease (NMD) [11] that results in progressive respiratory insufficiency as evidenced by a progressive decline in the FVC producing the typical restrictive respiratory pattern [11, 27]. Our findings indicate that advancing age, which is associated with progressive weakness and reduced FVC, was therefore expected. In fact, the trajectory of longitudinal changes in FVC changes in patients with DMD has been characterized by 3 stages as follows; 1) FVC rises annually during the ambulatory stage related to childhood growth; 2) FVC plateaus and remains stable during the early non-ambulatory stage; and 3) FVC declines progressively during the late non-ambulatory stage [5, 6] related to progressive neuromuscular weakness. The mean ± SD for age of our patients with DMD prescribed NIV was 20.1 ± 6.7 years which would fall into stage 2 or stage 3.

In patients with DMD, NIV is typically indicated for abnormal sleep studies, and/or FVC% predicted less than 50% predicted and/or maximal inspiratory pressure (MIP) < 60 cmH_2_O and/or awake baseline oxygen saturation < 95% or pCO_2_ > 45 mmHg [5, 6]. Thus, our patients with DMD prescribed NIV were quite advanced in their disease trajectory and as a result had an observed lower FVC% predicted values based on the natural history of DMD. Notwithstanding, we observed that the factors that were significantly associated with lower FVC% predicted included older age, presence of scoliosis, no deflazacort prescription and use of in-ex suffator in DMD patients using NIV.

There have been previous studies to date that have examined the association between clinical factors and lung function in individuals in DMD. Mayer et al. prospectively studied pulmonary function in 44 patients with DMD aged 5–24 years. Their group demonstrated that FVC and PEF were stable between the ages of 10–18 years and then decreased with increasing age as a near linear decline of 5% points/year from age 5–24 years similar to our study. They did not find an association between FVC and ambulation status and steroid treatment [15]. Similarly, Khirani et al. retrospectively evaluated the pulmonary function of 28 patients with DMD (aged range 6–19 years). This group found that gastric pressure during cough, sniff nasal inspiratory pressure (SNIP) and FVC% predicted significantly declined with age [28].

Presence of scoliosis was another factor that was significantly associated with lower FVC% predicted in our cohort. This is expected because the presence of scoliosis is a marker of more severe restrictive lung disease in all humans. The progression of scoliosis also impairs the action of the diaphragm by increasing diaphragmatic load and abnormal chest wall movement [29]. Scoliosis is a frequent complication in patients with DMD especially in the non-ambulatory stage [4, 30]. The worsening of scoliosis can cause a significant impact on the respiratory system resulting in reduced respiratory reserve [4, 31, 32]. Moreover, the diffuse respiratory muscle weakness causes less potential to resist the skeletal imbalance as a result it can progress more rapidly and easily overwhelm their respiratory muscle function leading to respiratory failure [33]. Hsu et al. demonstrated that in patients with DMD whose spinal curves exceeded 40 degrees, vital capacity is decreased by approximately 12 to 16% [30]. Previous retrospective cohort study revealed that spinal surgery for DMD scoliosis improved the FVC in surgical group in terms of scoliosis correction, delayed the reduction of FVC and consequently extended the survival rate compared to non-surgical group [34]. Therefore, routine orthopedic follow up for patients with DMD is paramount to prevent or delay scoliosis management which can lead to further respiratory function compromise [5].

Glucocorticoid prescription has gained traction to treat DMD patients as treatment to reduce inflammatory muscular destruction related to abnormal dystrophin protein [35–37]. In this context, glucocorticoids should similarly reduce inflammation induced muscular destruction in the skeletal walls of the chest wall. We found deflazacort prescription was associated with higher FVC% predicted in individuals with DMD using NIV. Based on our cohort, the individuals with DMD in SickKids having deflazacort (92.3%) had better lung function than the individuals with DMD having deflazacort in RCHSD (46.2%) and UCSD (15.0%), respectively. Previous studies have similarly reported that long-term glucocorticorsteroid treatment preserved spirometric parameters including FVC, FVC % predicted, FEV_1_, FEV_1_% predicted, PEF and PEF % predicted in patients with DMD over the second decade of life [11, 14, 38]. Sawnani et al. retrospectively examined PFT between glucocorticoid-treated patients with DMD and non-glucocorticoid-treated patients with DMD [14]. They found that the peak FVC% predicted was higher and achieved the peak FVC% at an earlier age in glucocorticoid-treated patients compared to non-glucocorticoid-treated patients and rates of decline for both groups varied with age [14]. Similarly, McDonald et al. prospectively studied the changes in PFT in 322 glucocorticoid-treated patients with DMD > 1 year and 53 non-glucocorticoid-treated patients with DMD. This multicenter study demonstrated that glucocorticoid-treated patients had higher peak absolute FVC and PEF values with later onset of decline and had slowed rate of pulmonary decline as measured by FVC% predicted [38]. However, these studies did not mention whether individuals with DMD were prescribed NIV. Recently, another retrospective study of adolescents with DMD initiating NIV over 10 years has been published to describe PFT changes with NIV between glucocorticoid users and non-glucocorticoid users. They showed that long-term glucocorticoid DMD users have lower lung function and faster decline, which slows following NIV initiation [18].

Cough function is important to augment airway clearance and prevent respiratory infection [39]. Impaired cough effort is common in the non-ambulatory stage of DMD causing atelectasis, pneumonia, and progression to respiratory failure [5, 6]. Prescription of an in-ex sufflator for chronic therapy is recommended when FVC < 50% predicted, cough peak flow (CPF) < 270 L/min and/or maximal expiratory pressure (MEP) < 60 mmHg [5, 6]. We found in our study that use of an in-ex sufflator was significantly associated with a lower FVC% predicted [39, 40]. This is likely because the use of an in-ex sufflator is a marker of more severe clinical disease.

Our study demonstrates marked variation in healthcare service utilization and deflazacort prescription across the three centers. In addition, in univariate analysis, we found that the institution was a factor that was significantly associated with FVC% predicted. While this factor was not associated following multivariate analysis, the observed differences in FVC should nonetheless highlight the differences in clinical practice, healthcare service utilization, resources, education, and family social support structures that may impact the progression of NMD [41]. Furthermore, the timing of NIV initiation may also be driving the differences in FVC% predicted that we found in our study. Our study underscores the significance of planning future multicenter prospective large-cohort studies. These studies will play a pivotal role in delving deeper into factors associated with the progression of DMD. This includes an exploration of elements influencing FVC% predicted and the intricate interplay with the timing of NIV initiation. Undoubtedly, adopting a multicenter approach would enhance the overall the generalizability and applicability of our findings.

There are some notable limitations to our study. First, it is a retrospective review, which leads to constraints in the available clinical data and pulmonary function parameters, including MIP, MEP, SNIP, and CPF. The three institutions had varying availability of specific pulmonary function tests such as CPF versus MIP/MEP, highlighting the heterogeneity of clinical practice in DMD patient care. Secondly, the sample size was limited owing to the rarity of the disease and the selective inclusion of solely NIV-utilizing patient. Consequently, this posed a challenge in achieving adequate statistical power to comprehensively examine multiple factors in multivariate regression analysis. Thirdly, our cohort study identified variations in reference ranges and methodologies for estimating height in patients unable to stand. Specifically, SickKids measured either arm-span or ulnar length for children with DMD, while RCHSD and UCSD employed ulnar length for estimating height in both children and adults with DMD. Percent predicted values were obtained based on Global Lung Initiative normative data in SickKids while C-NHANES III reference values was used in RCHSD and UCSD. These might have an impact on FVC% predicted values of individuals with DMD across three centers. These variations in clinical practice highlight the inherent challenges associated with multicenter approaches in pulmonary research. Fourthly, we did not ascertain the duration and adherence of therapy to deflazacort, in-ex sufflator use and HFCWO which would have additionally allowed us to evaluate the association between disease progression and pulmonary function. Fifth, due to limitations in data availability, we were unable to determine the age at NIV initiation or its impact on pulmonary function decline. Assessing a therapy's clinical response can be misleading without first considering the subject's current stage of FVC decline. Monitoring changes in the rate of absolute FVC decline provides a more precise measure of the therapy's effectiveness [11]. Our results may not fully resolve this concern, highlighting the need for future well-designed prospective studies to address this. Additionally, we could not conclude whether patients with DMD who initiate NIV experience progressively lower lung function, as we did not collect longitudinal pulmonary function data after NIV initiation. These limitations underscore the importance of establishing multicenter patient registries for DMD patients following initiation of NIV, with the goal of collecting comprehensive longitudinal data.

Notwithstanding these limitations, it is important to recognize that respiratory failure continues to be the predominant cause of mortality in patients with DMD. Hence, the identification of factors correlated with the improvement of FVC% predicted could contribute significantly to enhancing both morbidity and mortality outcomes in individuals with DMD encompassing both pediatric and adult population. The insights gleaned from our cohort should serve as a valuable resource for clinicians in identifying patients at risk for decreased FVC% predicted. Moreover, these findings can inform the design of future prospective longitudinal studies aimed at examining pulmonary function longitudinally in individuals with DMD.

## Conclusion

In summary, our multicenter study revealed key factors that are associated with lower FVC% predicted in individuals with DMD using NIV. These factors included older age, presence of scoliosis, absent deflazacort prescription, and use of in-ex sufflator, respectively. A future, multicenter large cohort prospective study is essential to further investigate factors associated with FVC% predicted and their interaction with the timing of NIV initiation in individuals with DMD.

## Data Availability

All relevant data are contained within the paper and the supporting information files. The data supporting the findings of this study are available from the corresponding author, Rakesh Bhattacharjee, upon reasonable request. Due to the nature of the data set, variables that may identify subjects (such as date of birth) have been removed. Further inquiries can be directed to the corresponding author.

## Abbreviations

AT: Adenotonsillectomy
ATS: American Thoracic Society
Bi-level PAP: Bi-level positive airway pressure
BMI: Body mass index
CPF: Cough peak flow
DMD: Duchene muscular dystrophy
ED: Emergency department
ERS: European Respiratory Society
FEF_25%‐75%_: Flows at lower lung volumes or forced expiratory flow over the middle one half of the FVC
FEV_1_: Forced expiratory volume in 1-s
FVC: Forced vital capacity
HFCWO: High frequency chest wall oscillator
IQR: Interquartile range
kg: Kilograms
LRTI: Lower respiratory tract infection
m: Meters
MEP: Maximal expiratory pressure
MIP: Maximal inspiratory pressure
NIV: Non-invasive ventilation
NMD: Neuromuscular disease
pCO_2_: Partial pressure of carbon dioxide
PCF: Peak cough flow
PEF: Peak expiratory flow
PFT: Pulmonary function test
PSG: Polysomnogram/ polysomnography
RCHSD: Rady Children’s Hospital, San Diego, CA, USA
SD: Standard deviation
SDB: Sleep-disordered breathing
SickKids: The Hospital for Sick Children, Toronto, Canada
SNIP: Sniff nasal inspiratory pressure
UCSD: University of California Health, San Diego, CA, USA.
VAPS: Volume-assured pressure support
